# Canalised and plastic components of melanin-based colouration: a diet-manipulation experiment in house sparrows

**DOI:** 10.1038/s41598-022-21811-2

**Published:** 2022-11-02

**Authors:** Agnieszka Gudowska, Katarzyna Janas, Justyna Wieczorek, Olga Woznicka, Przemysław M. Płonka, Szymon M. Drobniak

**Affiliations:** 1 Institute of Environmental Sciences, Faculty of Biology, Jagiellonian University, Kraków, Poland; 2 Institute of Systematics and Evolution of Animals, Polish Academy of Sciences, Kraków, Poland; 3 Museum and Institute of Zoology, Polish Academy of Sciences, Warsaw, Poland; 4 Department of Biophysics and Cancer Biology, Faculty of Biochemistry, Biophysics and Biotechnology, Jagiellonian University, Kraków, Poland; 5 Department of Cell Biology and Imaging, Institute of Zoology and Biomedical Research, Jagiellonian University, Kraków, Poland; 6 Ecology & Evolution Research Centre; School of Biological, Environmental & Earth Sciences, University of New South Wales, Sydney, Australia

**Keywords:** Ecophysiology, Evolutionary ecology

## Abstract

Whether melanin-based plumage colouration accurately reflects a bird’s quality is still controversial. To better understand potential mechanisms behind the observed variation in plumage colouration, we shifted our attention from a high-level expression of colour to low-level physiological phenomena by targeting the microstructure and pigment content of the feather. In a well-studied model system, the house sparrow (*Passer domesticus*), we combined an experimental manipulation of birds’ physiological condition and availability of resources that are key to the production of the studied colouration (phenylalanine and tyrosine (PT). We found that feathers from sparrows fed with the control diet had noticeably lower values of brightness, suggesting a higher quality of the ornamental “blackness” in comparison to those sampled from birds fed with a PT-reduced diet. Electron paramagnetic resonance (EPR) spectroscopy detected higher melanin concentrations in samples from the control than the PT-reduced group. Our multi-level analysis excluded mechanisms such as barbule density and melanosomes’ distribution, clearly pointing to the finest-level proxy of colour: the concentration of melanin in melanosomes themselves. Despite melanins being manufactured by birds endogenously, the efficiency of melanogenesis can be noticeably limited by diet. As a result, the birds’ plumage colouration is affected, which may entail consequences in social signalling.

## Introduction

Colourful feathers are among the most striking visible phenotypic traits in birds. They fulfil many crucial functions in a bird’s life cycle, from camouflage^1^, through thermoregulation^2,3^ to species recognition^4^. By far the most fundamental function of bird feather colouration is, however, sending visual signals. Hue, along with saturation and brightness communicate various aspects of individual quality^5–10^. Some colour ornament types are more likely to be costly to produce than others—and consequently may be more honest. Variation in costliness and honesty should stem predominantly from the mechanistic basis of an ornament: different colours are produced by different mechanisms which, through various underlying limitations, shape the overall cost of producing a signal^11^. Literature provides many attempts at experimentally testing the costs of colourful ornaments^12,13^—but they are often contradictory and ambiguous. Thus, we are far from understanding the proximate basis of honesty in most of such signals.

Melanin-based pigmentation is the most abundant and widespread form of colouration in animals, occurring in virtually all vertebrate clades, and particularly important as one of the key pigments providing colouration to bird feathers^14^. It occurs in two different forms based on two distinct pigments: eumelanin and pheomelanin. Eumelanin is perceived as black to dark brown, whereas pheomelanin produces reddish-brown and rusty shades^15^. To date, all examined melanin-containing feathers were shown to include both chemical variants, thus the reflectance spectrum of melanin-coloured feathers is a function of the ratio of eumelanin to pheomelanin^16^. Whether melanin-based plumage colouration accurately reflects a bird’s quality is still controversial^17–19^.

Melanins, contrary to pigments coming directly from diet, e.g., carotenoids, are synthesized by birds endogenously. Thus, unlike carotenoid-based colours, there is no direct link between access to pigments and expression of the colour^20^. The process of production and accumulation of melanins appears to be weakly sensitive to the environment and strongly heritable^19,21^. It leads to the suggestion that melanin ornaments are not condition-dependent and relatively cheap to produce, undermining the presumed honesty of melanin-based signals. However, melanin-based ornaments may vary both in the intensity of colour expression and in the area of plumage coverage^22^. All of these components can measurably depend on external and physiological factors. For example, plumage traits of house sparrow were affected by components of the diet they consumed during moult^18^. In the black-headed gull (*Chroicocephalus ridibundus*) the size of a melanin-based ornament (brown hood) was positively associated with bird condition, and, at the same time negatively correlated with the level of physiological stress^23^. It is therefore important to discover why different individuals deliver pheomelanins and eumelanins at varied concentrations, how they generate the final variability in the expression of the signal, and how the observed variation may bear appropriate signalling value.

Melanin synthesis is a highly complex process involving several feedback mechanisms and regulatory processes activated by e.g. hormones, neurotransmitters, growth factors, as well as receptor-independent mechanisms activated or modified by nutrients, micromolecules, microelements, pH, and many more^24,25^. Substrates needed to produce melanic ornaments are ultimately sourced from an animal’s diet and therefore, in case of external scarcity, their availability to colour-producing processes may be limited, or they may be differently allocated across other internal maintenance functions. A series of trace dietary minerals (e.g., Ca, Zn, Cu, Fe) act as critical regulatory factors in the biosynthesis of melanin^26–28^. Furthermore, because melanins are synthesized from aromatic amino acid precursors (directly from tyrosine or indirectly from phenylalanine), with thiol-containing substrates, such as cysteine, necessary for pheomelanogenesis, it is plausible that dietary access to those amino acids may restrict or enhance the concentration of melanin pigments in feathers^18,29^. Dietary aromatic amino acids are also biochemical precursors of several important neurotransmitters, which may additionally bind melanin-producing processes with other physiological functions^30^. To better understand potential mechanisms behind observed plumage variability within a species, it is necessary to focus research on dietary restrictions and physiologically limiting processes. Moreover, attention focused on a final product—commonly measured high-level expression of colour, namely hue, saturation, and brightness, should be extended to lower-level mechanistic processes. Low-level physiological phenomena, such as tissue microstructure and pigment content of the feather, may turn out to be crucial in understanding the mechanisms underlying externally visible variation in colouration.

To achieve this, we have designed a factorial experiment and analysed melanin-based colouration at multiple levels of mechanistic complexity. Physiological limitation in the underlying biochemical processes was achieved by introducing an immune challenge (which should affect the body condition axis of underlying pathways), and by dietary manipulation, where birds were fed control, wholesome food or a diet with significantly reduced precursors essential for melanin synthesis. We combined colour reflectance measurements of the feather’s vane with spectroscopic methods to determine the spatial density of melanosomes embedded in keratin, as well as to identify and quantify melanins enclosed in pigment granules. Moreover, because the feather growth rate during melanogenesis and feather microstructure may affect colouration, we measured feathers development rate and barbs’ density. This novel combination of methods was applied to a model species—the house sparrow (*Passer domesticus)* and its black bib expressed by males. The bib is a conspicuous trait that may indicate an individual’s body condition and define its dominance status^31,32^. If colouration of melanin-based feathers is a condition-dependent trait, we expected the experimental manipulations to affect the spectrophotometric characteristics of the bib feathers. We expected the two underlying parameters—melanosomes density and melanosome pigment concentration—to be affected by the limiting factors in parallel, providing a mechanistic basis for externally observed changes in feather pigmentation.

## Results

### Food consumption and body mass

**Figure 1:**
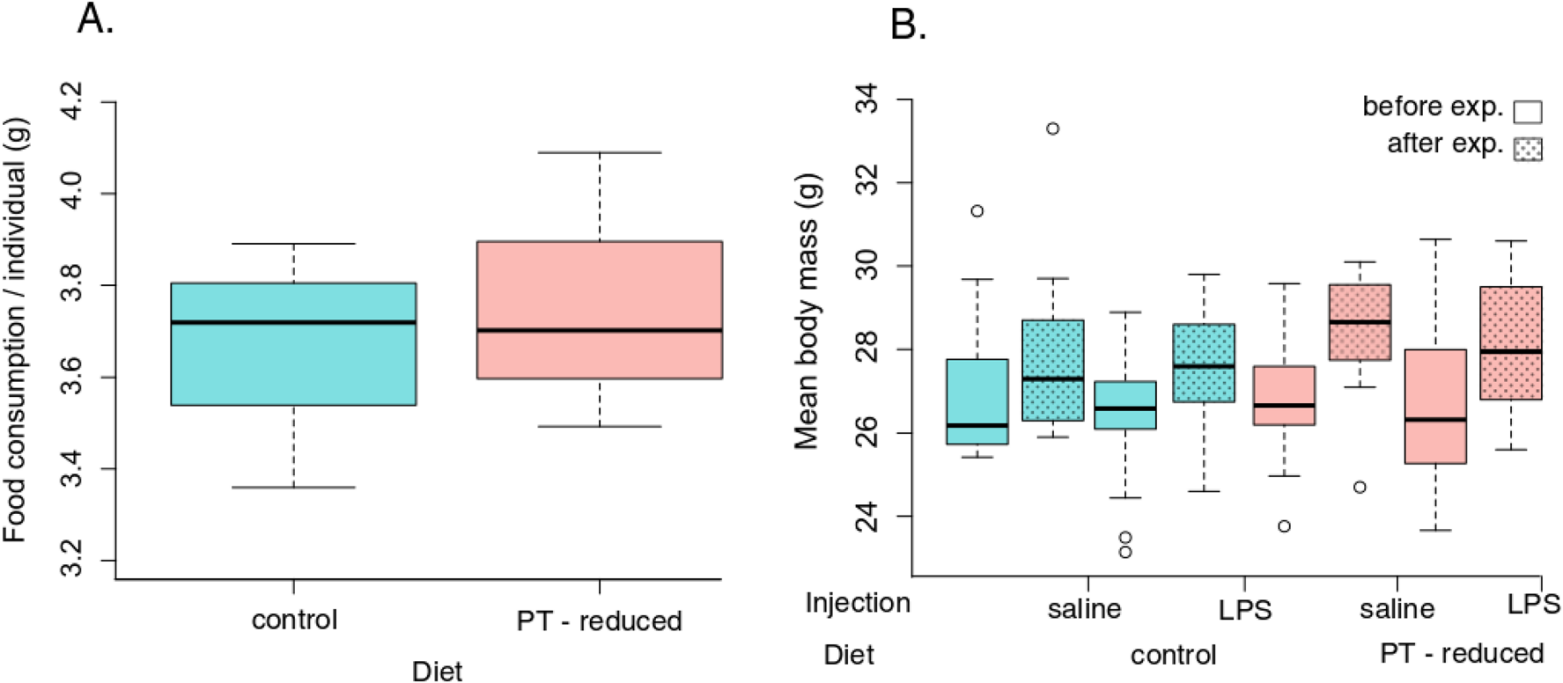
Food consumption and body mass of house sparrow. Food consumption (g) per individual in control and PT-reduced diet (phenylalanine and tyrosine at 42% of their level in the control diet; **A**), body mass of house sparrows before and after the experiment (**B**). Control males were injected with saline vehicle, whereas immune challenged (LPS) birds were injected with lipopolysaccharide.

Food consumption rates (averaged over three days) exhibited no significant differences between birds fed with the PT-reduced diet and those on the control diet (Friedman test, X^2^ = 0.33, df = 1, p = 0.56, Fig. 1A).

All birds gained mass over the course of the experiment, regardless of the treatment (linear mixed effect model: F_1,60_ = 95.72, p < 0.001; Fig. 1B,S3). Interestingly, individuals that have been kept on the PT-reduced diet gained more mass than birds fed with the PT-control diet (linear mixed effect model, time of measurement × diet type interaction, estimate ± SE = 0.62 ± 0.26; F_1,60_ = 5.56, p = 0.02, S3). Statistically non-significant interactions (time × immune challenge; diet × immune challenge) were removed from the final model.

### Feather brightness

**Figure 2:**
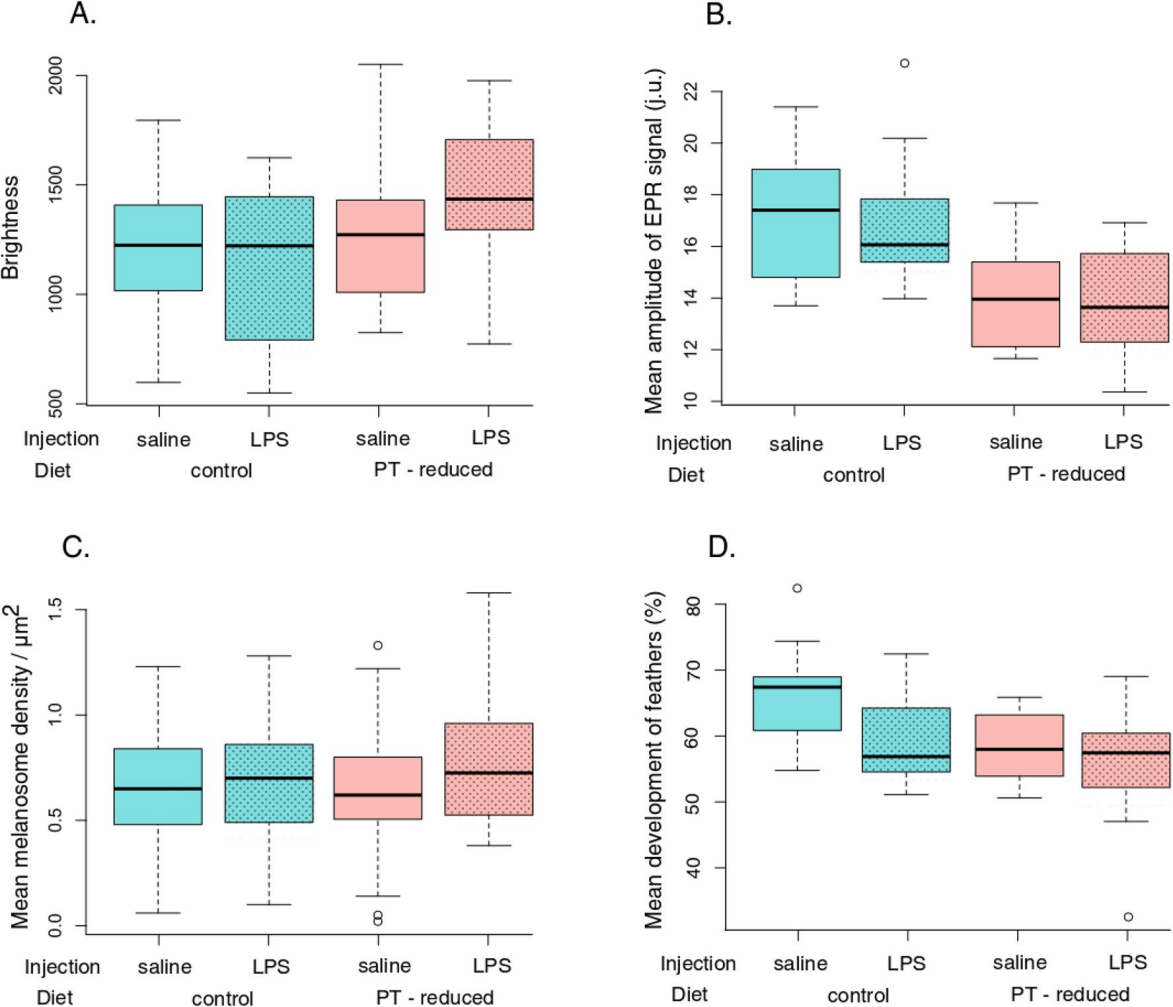
Feather parameters in different experimental treatment. Boxplot of mean brightness (**A**), mean amplitude of the EPR signal (**B**), mean melanosome density per µm^2^ (**C**), and mean development of feathers (**D**) of bib feathers of house sparrow fed control or PT-deficient diet and immune challenged (LPS) or injected by saline vehicle (control group).

The brightness of the bib feathers, expressed as a sum of the reflectance values over all wavelengths of a spectrum, varied with the diet but not with the immune challenge (Fig. 2A). Sparrows fed with control diet had lower values of brightness than individuals kept on a PT-reduced diet (two-way ANOVA: estimate ± SE = 183.36 ± 91.40; *F*_1,58_ = 4.48, *P* < 0.05, S4). Differences in brightness were not caused by density of feathers (two-way ANOVA: estimate ± SE = 165.05 ± 226.35; *F*_1,58_ = 0.53, *P* = 0.47, S4). Statistically non-significant interaction (diet × immune challenge) was removed from the final model.

### Melanin content

The overall level of melanin pigmentation was significantly affected by the bird’s diet, but not the LPS exposure (immune challenge by lipopolysaccharide) or the interaction of the two factors (Fig. 2B). Samples from sparrows fed with control diet revealed EPR spectra with higher mean peak-to-peak amplitudes compared with those from the group fed with PT-reduced diet, signalling a decreased concentration of melanins when the two exogenous amino acids were deficient (two-way ANOVA: estimate ± SE = − 3.15 ± 0.59; *F*_1,53_ = 29.07, *P* < 0.001, S5). Interestingly, no significant difference in pigment content was observed in response to the immunological challenge (two-way ANOVA: estimate ± SE = 0.24 ± 0.60; *F*_1,53_ = 0.09, *P* = 0.77, S5). Differences in melanin content were not caused by varying densities of feather tissue as this covariate was controlled for in our models (two-way ANOVA: estimate ± SE = − 0.10 ± 1.45; *F*_1,53_ = 0.47, *P* = 0.49, S5). No interaction observed between both independent factors (dietary and immunogenic) meant that, when applied simultaneously, the two experimental factors did neither amplify nor reduce their respective overall effects on the quantity of melanin in the bib feathers.

### Melanosome density

In both experimental manipulations, namely diet and immune challenge, feathers from the control and experimental group were characterised by similar densities of melanosomes (two-way ANOVA: estimate ± SE = 0.03 ± 0.06, *F*_1,58.7_ = 0.28, *P* = 0.60 for the diet manipulation, and estimate ± SE = − 0.10 ± 0.06, *F*_1,58_ = 2.87, *P* = 0.10 for the immune challenge, Fig. 2C; S6). The intraclass correlation coefficient indicated a satisfactory technical repeatability of 0.65, supporting the robustness of our assumption that melanosomes are distributed uniformly across the black portion of the feather.

### Feather development and density

Both the diet and immune challenge affected the feather development rate measured as the ratio of the developed part of the feather to its total length (Fig. 2D). Specifically, after three weeks from the beginning of the experiment, feathers were less developed (grew slower) in birds fed the PT-reduced diet in comparison to birds fed the control diet (two-way ANOVA: estimate ± SE = − 0.05 ± 0.02, *F*_1,59_ = 8.08, *P* = 0.006, S7). LPS-treated house sparrows had less developed feathers than the control individuals (two-way ANOVA: estimate ± SE = 0.04 ± 0.02, *F*_1,59_ = 6.11, *P* = 0.02, S7). Statistically non-significant interaction term (diet × immune challenge) was removed from the final model. Despite different rates of feather development between treatments, mean feather mass of growing feathers did not differ according to either experimental manipulation (two-way ANOVA: estimate ± SE = − 0.01 ± 0.02, *F*_1,59_ = 0.20, *P* = 0.66 for the diet manipulation and estimate ± SE = 0.001 ± 0.02 *F*_1,59_ = 0.002, *P* = 0.96 for the immune challenge).

The density of the feather’s vane (more precisely: numbers of barbs) was not affected by either the quality of the provided food and the physiological stress introduced by the immune challenge (two-way ANOVA: estimate ± SE = 0.05 ± 0.05, *F*_1,59_ = 0.99, *P* = 0.32 for diet and estimate ± SE = 0.04 ± 0.05 *F*_1,59_ = 0.61, *P* = 0.44 for immune challenge, S8).

## Discussion

**Figure 3:**
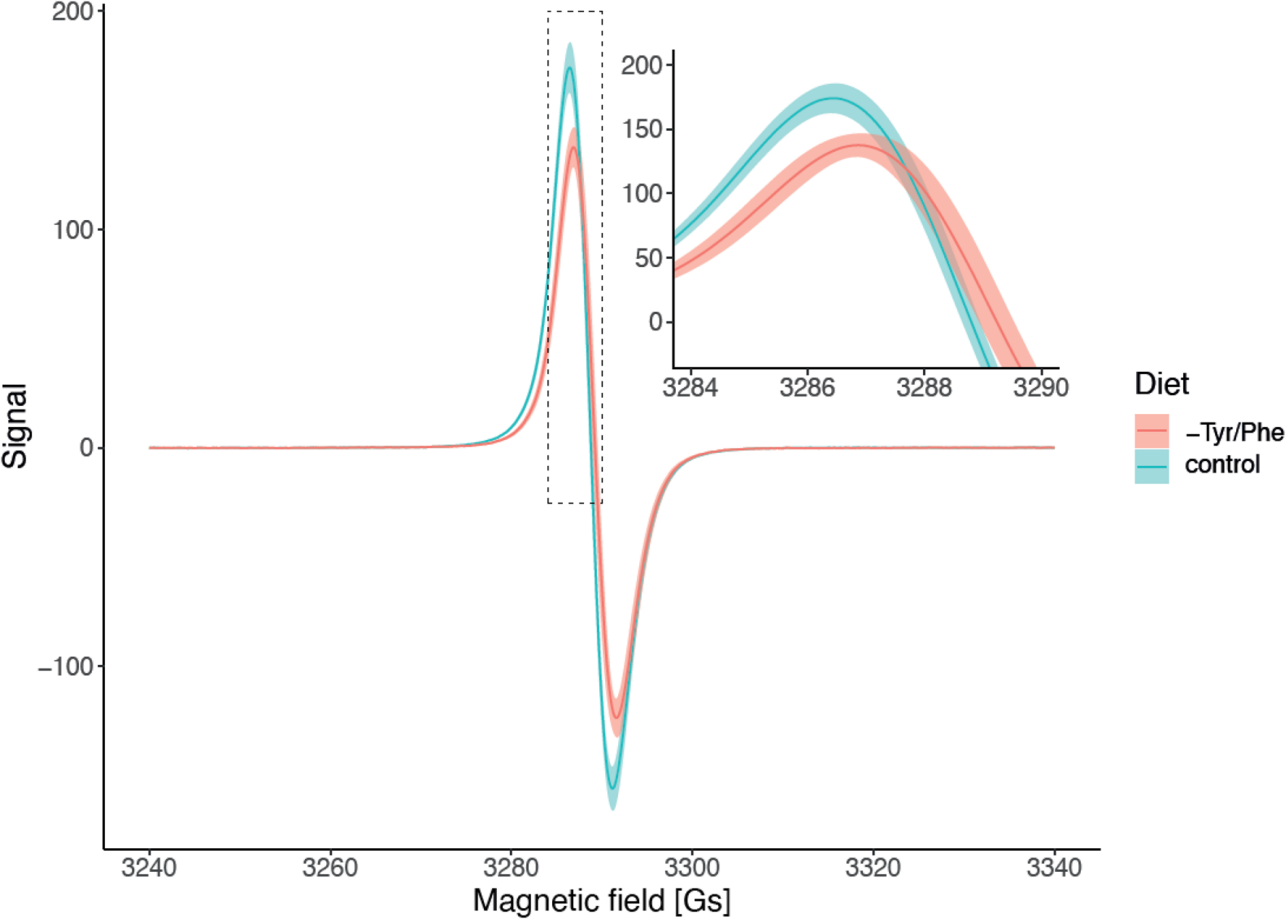
Examples of electron paramagnetic resonance spectroscopy (EPR). The EPR showing quality and quantity of melanin pigments in feather samples obtained from the bib of house sparrows. Each curve was calculated as mean EPR signal from all individuals coming from the same treatment.

Comprehensive approaches to animal colouration akin to that used in our paper are not common. Originality of our study stems from a hierarchical approach of investigating the melanin-based colouration itself (i.e., the perceived colour trait) and dissecting its variation into components acting at the finer mechanistic scales. Framing our study in an experimental context created a powerful result that demonstrates the strength of a multidisciplinary, holistic approach. Starting from the high-level colour expression traits, we found that the brightness of the bib feathers varied according to the diet treatment but not with respect to the immune challenge. Feathers plucked from sparrows fed with the control diet expressed noticeably lower values of brightness, suggesting an increased absorption of visible light due to more efficient melanin production and its deposition, in comparison to those sampled from birds fed with the PT-reduced diet. This interpretation was confirmed by analysing the low-level phenomena, using EPR spectroscopy and TEM imaging. We detected higher melanin concentrations in samples from control sparrows than in those collected from the PT-reduced group (Fig. 3). These results contradict the idea that melanins allow animals to produce colour traits independently of body condition or the environment in which they live^33^.

A considerable number of previous studies have shown that variation in melanin-based colouration is predominantly due to genetic factors. A few candidate genes were identified as involved in this type of pigmentation—the melanocortin 1 receptor (*Mc1R)*, agouti-signaling protein (*ASIP*), tyrosinase (*TYR*), follistatin (*FST*^24,33–35^). Thus, an overall picture is that melanin-based traits are moderately to strongly heritable (0.53 ≥ *h*^2^ ≥ 1), indicating that such traits should be strongly canalized and under weak environmental control^33,36^. Such pattern would constrain the possibility of melanin-based ornaments to signal phenotypic quality not strictly linked to genotype. Nevertheless, evidence of environmental-induced and condition-dependent variation in melanin-based ornaments is successively growing and needs updating^12,37^. Notably, a meta-analysis comparing melanin- and carotenoid-pigmented ornaments in birds did not detect any significant difference in their condition dependence^37^. Moreover, empirical evidence suggests that melanocortin signalling may be an independent modulator of melanin pigmentation, leaving enough space for condition- and environment-dependent processes to occur and contribute honest information to melanin-based ornaments^38^. In line with this, our results showed that suboptimal diet impaired melanin-based plumage colouration, which may have further consequences for an individual, e.g., in social signaling. Similarly to our study, Poston et al., (2005) have found that reduction of melanin precursors in diet produced lighter bib feathers in house sparrows. The aforementioned results are in contrast with another study on house sparrow males where the size and brightness of their melanin-based throat badges were not affected by nutritional stress^39^. On this basis it can be concluded that throat badges may be sensitive only to specific components of the diet and not to general food restriction. The prediction is supported by study conducted on zebra finches (*Taeniopygia guttata*) where the expression of ornamental melanin colouration depended on acquisition of minerals (e.g. calcium; 19). In blackcaps (*Sylvia atricapilla*), darkness of melanin-based ornaments positively correlated with nutritional condition across seasons^40^, however, it is not known whether the effect was caused by general diet restriction or restriction of a specific diet component. In addition to the components of the diet, other condition-related factors (e.g. disease state, hormonal control) were reported to affect melanin pigmentation^41–44^. Furthermore, darker, more eumelanic individuals are hypothesized to be more resistant to stress than lighter conspecifics^21^. Study on the expression of a melanin-based signal: the well-known black stripe of the great tit (*Parus major*) and the red-legged partridges (*Alectoris rufa*) indicated that such signal can be at least partially determined by oxidative stress^38,45^. In contrast to many species with exaggerated melanin ornaments only in males, the ornaments can predict a component of reproductive performance also in females of Northern Flicker (*Colaptes auratus*;^46^). Therefore, mate choice based on the expression of melanin-based colouration may contain multiple messages and provide not only indirect genetic benefits (offspring of higher genetic quality), but also honest information and direct material benefits (better territory quality, parental care; 18).

Melanin is synthesized and stored in melanosomes, thus darker plumage can be caused by the deposition of larger amounts of melanin^47^, by increasing the density of melanosomes, and/or modifying feather microstructure^17^. Field et al.^48^, quantitatively demonstrated a link between feather melanosome concentration and feather colours in fossilised and extant feathers. In our study, the response of melanin concentration to experimental manipulation was not reflected in a parallel trend involving the density of melanosomes. Feathers collected from both control and experimental birds were characterized by similar densities of melanosomes. The lack of such dependency, with a concurrent modification of the overall melanin content, suggests that melanosomes in manipulated birds differ in melanin content. Within individual melanosomes, the concentration of melanin may vary few fold^49^. It emphasizes that researchers should be more cautious when assigning variation in melanin-based plumage colouration to melanosome density alone. Although concentration of melanin is critical in the production of colour, other components of feather may also contribute. For example, melanin ornamentation in great tits (*Parus major*) was darker when the feathers were composed of more numerous barbules and thicker barbs^50^. Similarly, black-capped chickadees (*Poecile atricapillus*), affected by the avian keratin disorder, and zebra finches (*Taeniopygia guttata*), subjected to unpredictable food, had less barbules and, in effect, duller feathers than control birds^17^. These results clearly indicate that feather microstructure plays a role in melanin-based colour production and may be condition-dependent. Additionally, our study suggests some degree of modularity and independence among those mechanistic processes underlying the expression of colour. On the one hand, diet manipulation did result in a reduction of melanin concentration. In contrast, both experimental treatments—the PT-reduced diet and the immune challenge triggered by LPS injections—seemed to have slowed down the rate of feathers’ growth, without any noticeable effects on the melanin-related parameters of feather pigmentation. The biochemical pathway that forms melanin begins with the amino acid tyrosine sourced from diet or synthesized from phenylalanine^15^. However, the same amino acids are used to a number of biochemical pathways, endogenous signalling, and hormonelike bioregulators e.g. production of thyroid or coping hormones^24,51^. Thus, shortage of PT in a bird’s diet can be involved not only in the quality of melanin ornaments, but also modulate behavioural expression by reducing learning abilities, increasing neophobia and aggression^30^. Ability to optimize foraging efficiency over time appears to be an accurate signal of health and condition of individuals.

Apart from the differences in melanin concentration, the colouration variability of melanin ornaments in birds is, in part, a function of the ratio of the two types of melanins (eu- to pheomelanin), present in the feathers^16^. Moreover, each chemical form of melanin potentially conveys different information about physiological costs. Pheomelanogenesis is more costly in terms of its impact on the oxidative stress levels of an individual, compared to eumelanogenesis, because pheomelanin synthesis occurs when thiol-containing compounds (mainly l-cysteine and glutathione) are present above certain levels in melanocytes^52^. Thus, it is important to investigate the content of both melanin types in an ornament^53,54^. EPR measurements in our study not only confirmed the presence of melanin paramagnetic polymers in bib feathers, but also enabled us to identify that the dominant melanin in male sparrows’ bib feathers is eumelanin. In the measured samples, a clear hyperfine structure, typical for the presence of phaeomelanin (resulting from the differences in the chemical characteristics of pheomelanin-like paramagnetic centres) was not detected. This simplifies the interpretation of our results, but also emphasizes that future studies looking at mechanistic underpinnings of melanin-based colouration should attempt to decompose the underlying mechanisms into those resulting from eumelanin versus phaeomelanin (especially because the dominant form of melanin might potentially mask the presence of the other in terms of produced colouration, making methods such as EPR or HPLC the most practical ways of detecting these pigments in feathers).

Together, our results strongly suggest that, despite melanins being manufactured by birds endogenously, the efficiency of melanogenesis can be noticeably limited by diet, particularly by depleting the bird from the essential precursors of melanins—namely, the amino acids phenylalanine and tyrosine. Therefore, expression of the melanin ornaments also depends on nutritional conditions during moult which suggests that melanin may be an honest advertisement of individual quality. Thus, our results are in line with the study based on 21 tested species, where the authors found support for a positive relationship between condition and melanin-dependent plumage, with darker or larger areas of melanin-based plumage signalling better individual quality and health status^12^. Additionally, in our study, we demonstrate a simple and high-throughput method that can be used to estimate the relative concentrations of paramagnetic colour-producing substances in feathers. In contrast to more *widely used analytical methods, such as* high-performance liquid chromatography (HPLC), EPR spectroscopy does not require multistep extraction of the pigments, and feather samples can be reused for other measurements.

## Materials and methods

### Birds and housing

62 males and 8 females of house sparrows were caught with mist nets in September and October 2019 in several sites in Kraków, Poland. Before releasing them to the outdoor aviary located on the campus of the Jagiellonian University, Kraków, Poland, each bird was weighed and banded with a metal band. The aviary measured 3.5 m in width, 10.0 m in length, 2.5 m in height, and was outfitted with trees, bushes, perches, wooden shelters, a water source, and food dishes. Initially, birds were maintained with water and a mixture of seeds: wheat, barley, millet, and sunflower seeds, provided ad libitum. Additionally, they had access to sand with shells and sepia.

### Experimental design

After a few weeks of acclimation to captivity, the aviary was divided into two separate parts (3.5 × 5 m): aviary no. 1 (A1) and aviary no. 2 (A2). At the same time male individuals were assigned to two crossed experimental treatments, ensuring that in each aviary birds originated from all sampled populations. The experiment comprised of two different treatments conducted simultaneously—one designed to simulate a deficiency in an environmental factor influencing colouration (the quality of available food), the other—to introduce physiological stress and facilitate trade-offs in the allocation of resources limited by the first treatment (an immune response induced by a bacteria-derived compound, S1).

The dietary manipulation was achieved by feeding one group of birds with a low-quality protein food (diet reduced in exogenous amino acids, namely phenylalanine and tyrosine content, which are precursors essential for melanin synthesis; PT-reduced diet), and the other one with a wholesome diet (control diet). At the same time, two levels of immune challenge were achieved within each dietary group, by injecting half of the birds with either lipopolysaccharide (LPS) from the cell wall of *Escherichia coli*, or a 0.9% saline vehicle (as a control). Four females were placed in each group of males to alleviate interspecific conflicts occurring in all-male sparrow flocks, but they did not take part in the experiments. After three weeks of experiment, birds housed in A1 were moved to A2, whereas birds from A2 were moved to A1.

### Immune challenge

Before receiving injections, birds were first weighed and then transferred from the outdoor aviary to the laboratory. 31 house sparrows (from both dietary groups) were injected intraperitoneally with 0.026 mg LPS (serotype O55:B5, Sigma-Aldrich) diluted in 0.1 mL of 0.9% saline vehicle, so that each bird received a dose of ca. 1 mg/kg body mass, which had previously been shown to induce sickness behaviour in another passerine, the white-crowned sparrow, *Zonotrichia leucophrys*^55^. 31 control males were injected with the same volume (0.1 mL) of 0.9% saline vehicle. All individuals were injected twice throughout the experiment with an interval of three weeks between the injections. Birds were always injected at the same time in the morning and early afternoon (between 9:00 am and 12:30 pm).

### Diet manipulation

During the six weeks of the experiment (S1), birds received synthetic diet ad libitum, which constituted of a mixture of protein (WPC80, free amino acids and whey protein isolate BiPRO GMP 9000 (Agropur Inc., Appleton, USA)), fats, carbohydrates, and fiber^30^. The ingredients were thoroughly mixed to produce small pellets (6 mm in diameter) that the sparrows consumed readily. The experimental diet had phenylalanine and tyrosine at 42% (*N* = 32) of their level in the control diet (*N* = 30)^30^. The food pellets were prepared by ZooLab (zoolab.pl/en/home, Sędziszów, Poland). Each bird was weighed before and after the experiment to monitor potential effects of diet on body mass of each animal. Following the experiment, during the next three consecutive days, the amount of food consumed by passerines within every 24 h (starting from 10 am each day to 10 am next day) was noted for both compartments of the aviary. Because of different numbers of individuals per aviary, an overall weight of food consumed in A1 and A2 was calculated per individual, respectively.

### Feathers sampling

Moult of the black bib feathers was stimulated at the end of the moulting period occurring in natural conditions in early November. At day 1 of the dietary/immunological experiment (S1) a small area of the bib (around 25 mm^2^) was plucked from each male sparrow held in A1. At day 2 the same procedure was performed on individuals from A2. The time difference is orders of magnitude smaller than the timescale of feather growth and hence it would not affect the results in any way.

Because the feather growth rate may differ during melanogenesis, with consequences for final colouration (if feathers grow at a faster rate, pigments may be deposited over a larger surface and therefore result in less intense colouration^56^, we measured the rate of feather development during the course of the experiment. After three weeks of the experiment, three feathers from the upper, central, and lower region of the previously plucked bib were plucked once again. The mass of the collected feathers was determined to the nearest 0.01 mg (XP26 Micro Balance, Mettler-Toledo, Greinfensee, Switzerland). The experiment was completed after six weeks after fully regrown and developed feathers from the bib and PC2 were sampled the second time (S1). Three feathers from the central part of previously plucked bib region were collected to perform transmission electron microscopy (TEM) imaging, whereas the feathers obtained from the rest of the regrown bib area were subjected to electron paramagnetic resonance (EPR) spectroscopy and feather microstructure analyses (greater spatial density of melanized barbs or barbules may affect colouration^17^.

### Feathers measurements

#### Reflectance measurements

An USB4000 spectrophotometer (range 300–700 nm) with the PX-2 Pulsed Xenon Lamp (Ocean Optics, Dunedin, FL, USA) and a bifurcated probe with 7 × 400 μm optical fibres, equipped with a permanently attached 3 mm long black collar, was used to quantify the brightness of the bib feathers collected at the end of the experiment. The measurements were taken with 90 ms integration time and the probe held at 90° to a feather’s surface. Calibration measurements of a Spectralon white standard (Ocean Optics. Largo, FL, USA) were taken every 15 min during measurements. The order in which the samples were measured was randomized in terms of belonging to the experimental group. From each sample (*N* = 62), seven feathers were chosen and stacked in one pile on a piece of black paper. Ten reflectance measurements were taken on each pile, avoiding distal, brighter parts of the feathers. The obtained spectra were averaged and smoothed in the package ‘*pavo*’^57^. Brightness was calculated as a sum of the reflectance values over all wavelengths of a spectrum, and its lower values were interpreted as those indicative of a more melanin-rich feathers (i.e., absorbing more light).

#### Feather development

**Figure 4:**
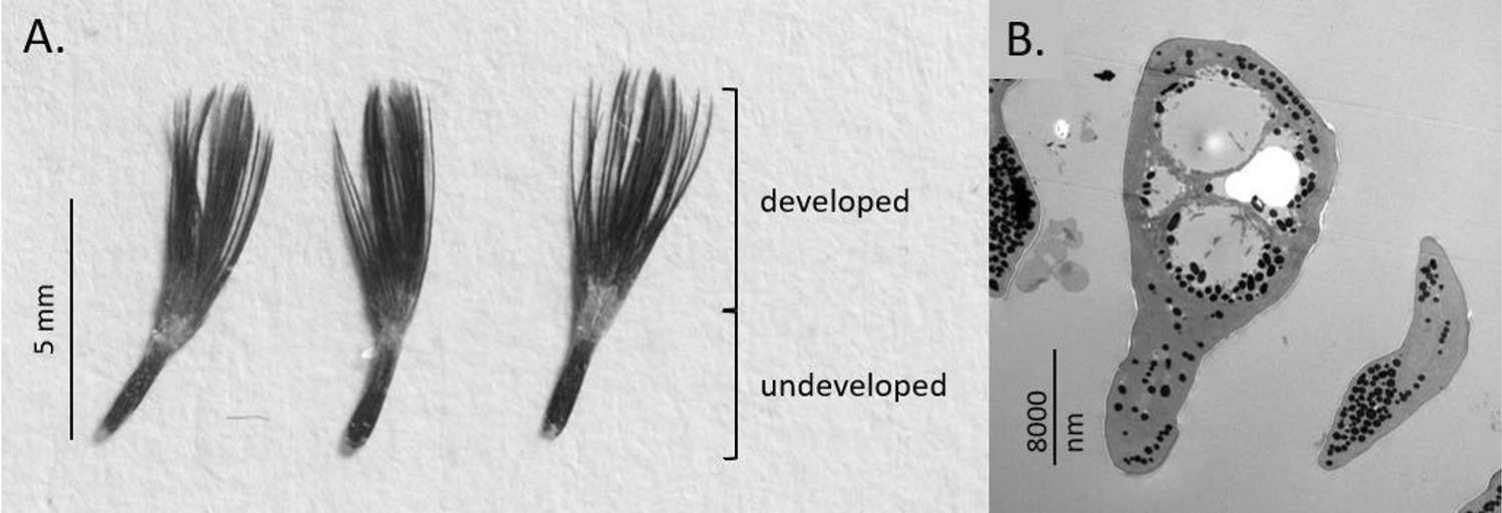
House sparrow feathers sampled from bib after three weeks of the experiment. Feathers during development (**A**), a TEM cross-sections of feather sampled from bib after the experiment (**B**).

Each feather (3 per individual; *N* = 62 individuals) was laid on a white card and covered by a microscope slide to flatten the naturally curved feathers. Digital photographs were taken using camera (Canon EOS 7D) and imported to ImageJ v1.52a Software (National Institutes of Health, USA). The lengths of fully developed and undeveloped (still in sheath) parts of each feather were measured. To estimate the degree of a feather’s development, the length of the developed part of the vane was divided by its total length (quill with rachis plus the developed vane, Fig. 4A).

#### Feather density

Barb density measurements were performed on the sampled regrown black bib feathers (*N* = 2–3 for each individual; *N* = 62 individuals), but because of their sparser structure we calculated the number of non-down (i.e., rigid) barbs on both sides of the vane, and divided this number by two (to obtain an average single-sided number of barbs) and then by the length of the rachis.

#### Melanosome density (TEM)

Feathers sampled from the bib of male sparrows (*N* = 62) were fixed for transmission electron microscopy (TEM) analysis in a mixture of 0.25 M sodium hydroxide and 0.1% Tween for 20 to 30 min on a bench-top shaker. Next, the feathers were treated with formic acid and ethanol in the ratio of 2:3 for 2.5 h and dehydrated twice for 20 min in 100% ethanol. Samples were embedded in a mixture of the PolyBed 812 resin (20 ml), DDSA (9 ml), NMA (12 ml) and DMP-30 (0.82 ml). Resin infiltration was gradual from 15% resin content in ethanol through 50%, 70% to 100% without alcohol. Each step lasted for 24 h. Then, the feathers were placed in silicone embedding moulds (Agar Scientific) and transferred to an oven. The polymerization proceeded at the temperature of 60 °C for 16 h. The epoxy resin blocks were then trimmed to get rid of excess resin. The surface of each block was prepared by its trimming, starting from the end of the feather, to approximately 5 mm using a glass knife. Next, ultrathin sections (70 nm) were cut with a diamond knife (DIATOME A. G., Berno, Switzerland) on a microtome (UC7, Leica, Wetzlar, Germany) and collected on single slot grids coated with a formvar film. The sections were then contrasted in uranyl acetate and lead citrate for 3 min. They were viewed and photographed with a transmission electron microscope (TEM) JEOL 2100HT (Jeol Ltd, Tokyo, Japan) for the purpose of investigating the number and density of the embedded pigment granules. For each individual three photographs of the cross-sections from a similar feather region were selected. Melanosome density was measured as the number of melanin granules observed in the barb cross-section divided by its area. Images were analysed using Adobe Photoshop (cross-sections area) and ImageJ (number of melanosomes, Fig. 4B).

#### Melanin content: electron paramagnetic resonance (EPR) spectroscopy

Quality and quantity of melanin pigments^58^ in individual feather samples obtained from the bib of house sparrows (N = 57) were characterized using a Varian E3 spectrometer (Varian, Sunnyvale, LA, USA) equipped with a rectangular resonance (TE 102) cavity. Five milligrams of feathers per individual were placed inside the Wilmad finger quartz dewar WG-816-Q (Rototec-Spintec GmbH, Griesheim, Germany). Prior to inserting the vessel into the resonance cavity of the EPR spectrometer, feathers were pressed down the quartz finger to a height of approximately 0.5 cm to ensure comparable volumes of each sample. Measurements were performed at room temperature, at X-band (9.26–9.27 GHz frequency), using the following parameters: magnetic field range 3240–3340 Gs, microwave power 1 mW, modulation frequency 100 kHz, modulation amplitude and time constant—5 Gs and 0.3 s for quantitative analysis, 1 Gs and 0.1 s for qualitative analysis. An EPR signal was recorded as its first derivative, averaged from three consecutive scans, lasting 160 s each (giving a total of 480 s of scan time per EPR spectrum). Then, the following parameters were measured*:* peak-to-peak amplitude, area under the microwave absorption curve (the integral intensity of the recorded signal) and linewidth of the EPR absorption curve (ΔH;^59^).

### Statistical analyses

Statistical analysis was performed in R (version 4.0.2,^60^) using a two-way ANOVA test, with bird’s diet (control vs. PT-reduced) and applied immune challenges (LPS vs. saline-injections) as the independent variables. The following parameters were used as the dependent variables: feathers reflectance (brightness), feather growth rate, feather density (number of barbs per mm), and melanisation level (expressed as the EPR spectrum amplitude measured in arbitrary units [a.u.]). The density of melanosomes was analysed by fitting a linear mixed-effects model. In this model, melanosome density was used as the dependent variable, with diet, immunological challenge, and slice ID as independent variables, and individual ID as a random-effect term. Additionally, to assess the reliability of measurements, the intraclass correlation coefficient (i.e., technical repeatability) was calculated. The models’ residuals were checked for normality and homoscedasticity. Mean food consumption per individual was analysed by the Friedman test. Body mass before and after the experiment was analysed by fitting a linear mixed-effect model. Body mass was used as the dependent variable, whereas diet, immunological challenge, and time as the independent variables, and individual ID as a random-effect term. The model included the following interaction terms: time × diet, time × injection, and diet × injection, and was reduced by removing the non-significant interactions. Results are reported with appropriate statistical tests and estimates (accompanied by standard errors) signifying relevant factor contrasts (relative to the reference group, which in all analyses was diet: control; injection: LPS, body mass: before experiment).

### Ethical note

All applicable national and institutional guidelines for the care and use of animals were followed. The research was performed under permit no. 25/2019 (with a supplementary permit no. 78/2020) from the 2nd Local Institutional Animal Care and Use Committee in Kraków.

## Supplementary Information

Supplementary Information.

## Data Availability

This original data analyzed in this paper has been uploaded to Figshare. It is available at 10.6084/m9.figshare.20391708.v1.
