# Supplementary material for: Canalised and plastic components of melanin-based colouration: a diet-manipulation experiment in house sparrows
Source: Sci Rep. 2022 Nov 2;12:18484. doi: 10.1038/s41598-022-21811-2 (PMC9630266; doi:10.1038/s41598-022-21811-2)
Supplement: Supplementary file 1 — Supplementary Information. [file 41598_2022_21811_MOESM1_ESM.pdf]

SUPPLEMENT

Supplementary methods

To better visualize the spectroscopic properties of two types of melanins found in birds, and verify the eumelanistic nature of sparrow's bib colouration, additional EPR measurements were performed using sparrow bib fathers and differently coloured chicken feather samples. The used feathers represented quantitative and qualitative variations in melanin content, and were compared with synthetic melanin standards and a paramagnetic reference.

A powder sample of 1,1-diphenyl-2-picrylhydrazyl (DPPH) was used as a paramagnetic reference, to determine the position of the feather melanin EPR signal, and indirectly - to determine the g-value. 3 small crystals of DPPH, approximately 2 mm in diameter each, were wrapped in parafilm (which did not disturb the free-radical signal) and placed inside the Wilmad finger quartz dewar. Additionally, powdered samples of dopa-auto-oxidation melanin and tyrosinase-catalysed cysteinyldopa melanin (both sealed in thin glass capillaries) served as synthetic equivalents of natural eumelanin and pheomelanin, respectively [1].

Next, samples of bib feathers were selected (each from every experimental group) and measured again, followed by a sample from a random male sparrow's tail, characterized by a less pigmented, much lighter, brownish hue, compared to the bib. 3 contour feather samples of black, reddish brown and white colourations originated from different chicken breeds (Sussex, Rhode Island Red, and Rhode Island White, respectively) represented control samples with different melanin composition and concentration. All samples weighed ~5 mg and were inserted inside the Wilmad finger quartz dewar in the same manner as during previous measurements. Black feather fragments of the Sussex (strain S-66, from the The National Research Institute of Animal Production, Balice n. Kraków, Poland) were used as an example of eumelanin-based black colouration, whereas dark, mahogany-coloured vanes from the Rhode Island Red (a gift from the Breene Acres Family Farm in West Greenwich in Rhode Island, USA), represented a pheomelanin-rich sample. White feathers obtained from the Rhode Island White chicken (strain A-33, from the The National Research Institute of Animal Production, Balice n. Kraków, Poland) were used as an amelanotic example and helped emphasize the difference in EPR signal quality in comparison to samples rich in melanin pigments [2,3]. Each EPR signal was averaged from 3 scans, lasting 160s each, and was recorded at room temperature, at X-band (9.26 GHz frequency), using the following parameters: magnetic field range 3240-3340 Gs, microwave power 1 mW, modulation frequency 100 kHz, modulation amplitude and time constant – 1 Gs and 0,1 s .

Supplementary results

A statistically significant, positive, low to moderate (R-square 0.2955 – 0.6367, $p<0.05$) linear relationships between mean EPR signal's amplitude values and its corresponding integral intensities were observed within each treatment group. At the same time, the values of EPR spectra linewidths remained relatively constant, and are unrelated to the shape of the recorded spectra. Therefore, the peak-to-peak height of the central feature of the spectra can be used as a sufficient approximation of the concentration of paramagnetic centres found in melanins [4]. The mean values of EPR amplitudes were used as a direct indicator of the melanisation level in feather samples.

Comparison between the EPR spectra of synthetic melanin analogues (DOPA melanin, cysteinyl-DOPA melanin) and differently coloured feather samples: black barbs and barbules of the Sussex chicken and sparrow's bib, reddish brown contour feathers of the Rhode Island Red, and control white samples of the Rhode Island White, helped visualize the spectroscopic differences between the presence of eumelanin and pheomelanin pigments, as well as between melanotic and amelanotic keratinous appendages (S2). These results further show that the EPR spectra recorded for sparrows black feathers are directly related to the paramagnetic centers found in melanins and not the free radicals formed in keratin.

The conducted spectroscopic analysis supports the assumption that the black patch located on the throat of a male house sparrow results from the presence of paramagnetic polymers – melanins [2]. The slightly asymmetric lineshape of EPR signals of similar linewidths (4.200 ± 0.063 [Gs]*)* and with no distinct hyperfine-splitting, resembles that of dopa melanins (S2), which are often used as a synthetic model of natural eumelanins. These features, along with the lack of the hyperfine splitting (typical for benzothiazine-based pheomelanins and their synthetic standard – cysteinyldopa melanin (S2)), strongly suggest that eumelanin is the dominant pigment responsible for the bib colouration. However, these results do not entirely exclude the presence of pheomelanins, which might be present in the investigated samples at a negligible level/below the detection level of the EPR spectrometer. Another possible explanation lies in the chemical structure of these polymers, namely the predominance of benzothiazole units, whose presence, compared to benzothiazine moieties, might result in a broadened signal and less pronounced splitting in the low-field part of the EPR spectrum [5–7]. DPPH provides a stable radical source with a well-defined g-value (2.0037 ± 0.0002). By assessing the position between the EPR signal of this free radical standard and the EPR spectra recorded for feathers samples and synthetic melanins, we were able to estimate the g-value of the paramagnetic centers detected in the sparrows bib and other feather samples, and prove that the recorded signals origin from the paramagnetic centers of natural melanins, embedded in the feathers keratin (S2).

REFERENCES

1. Wolnicka-Glubisz, A., Pecio, A., Podkowa, D., Plonka, P. M. & Grabacka, M. HGF/SF increases number of skin melanocytes but does not alter quality or quantity of follicular melanogenesis. *PLoS ONE* **8**, 1–11 (2013).
2. Barden, H. E. *et al.* Morphological and geochemical evidence of eumelanin preservation in the feathers of the Early Cretaceous bird, *Gansus yumenensis*. *PLoS ONE* **6**, (2011).
3. Strzelczak, G., Sterniczuk, M., Sadło, J. & Kowalska, M. EPR study of γ -irradiated feather keratin and human fingernails concerning retrospective dose assessment. **58**, 505–509 (2013).
4. Plonka, P. M. Electron paramagnetic resonance as a unique tool for skin and hair research. *Experimental Dermatology* **18**, 472–484 (2009).
5. Sealy, R. C., Hyde, J. S., Felix, C. C., Menon, I. A. & Prota, G. Eumelanins and Pheomelanins: Characterization by electron spin resonance spectroscopy. *Science, New Series* **217**, 545–547 (1982).
6. Pukalski, J. *et al.* Detection of a pheomelanin-like pigment by EPR spectroscopy in the mycelium of *Plenodomus biglobosus. Acta biochimica Polonica* **67**, 295–301 (2020).
7. Zadlo, A., Mokrzyński, K., Ito, S., Wakamatsu, K. & Sarna, T. The influence of iron on selected properties of synthetic pheomelanin. *Cell Biochemistry and Biophysics* **78**, 181–189 (2020).
8. Sealy, R. C., Hyde, J. S., Felix, C. C., Menon, I. A. & Prota, G. Eumelanins and pheomelanins: Characterization by electron spin resonance spectroscopy. *Science* **217**, 545–547 (1982).

SUPPLEMENTARY FIGURES AND TABLES

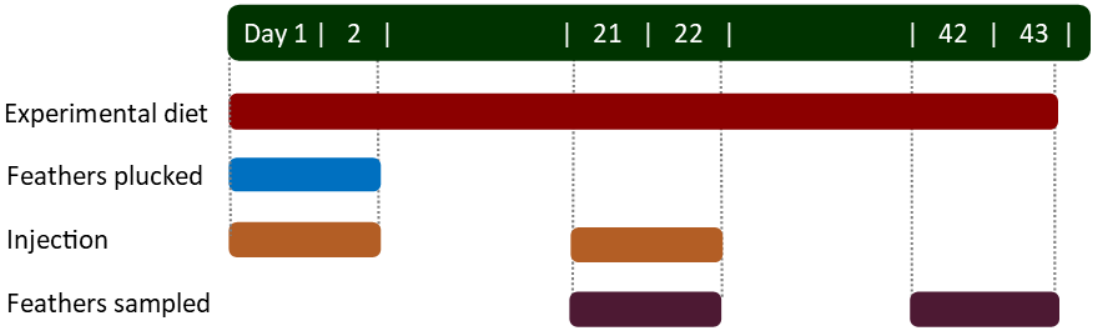

S1. Experimental timeline.

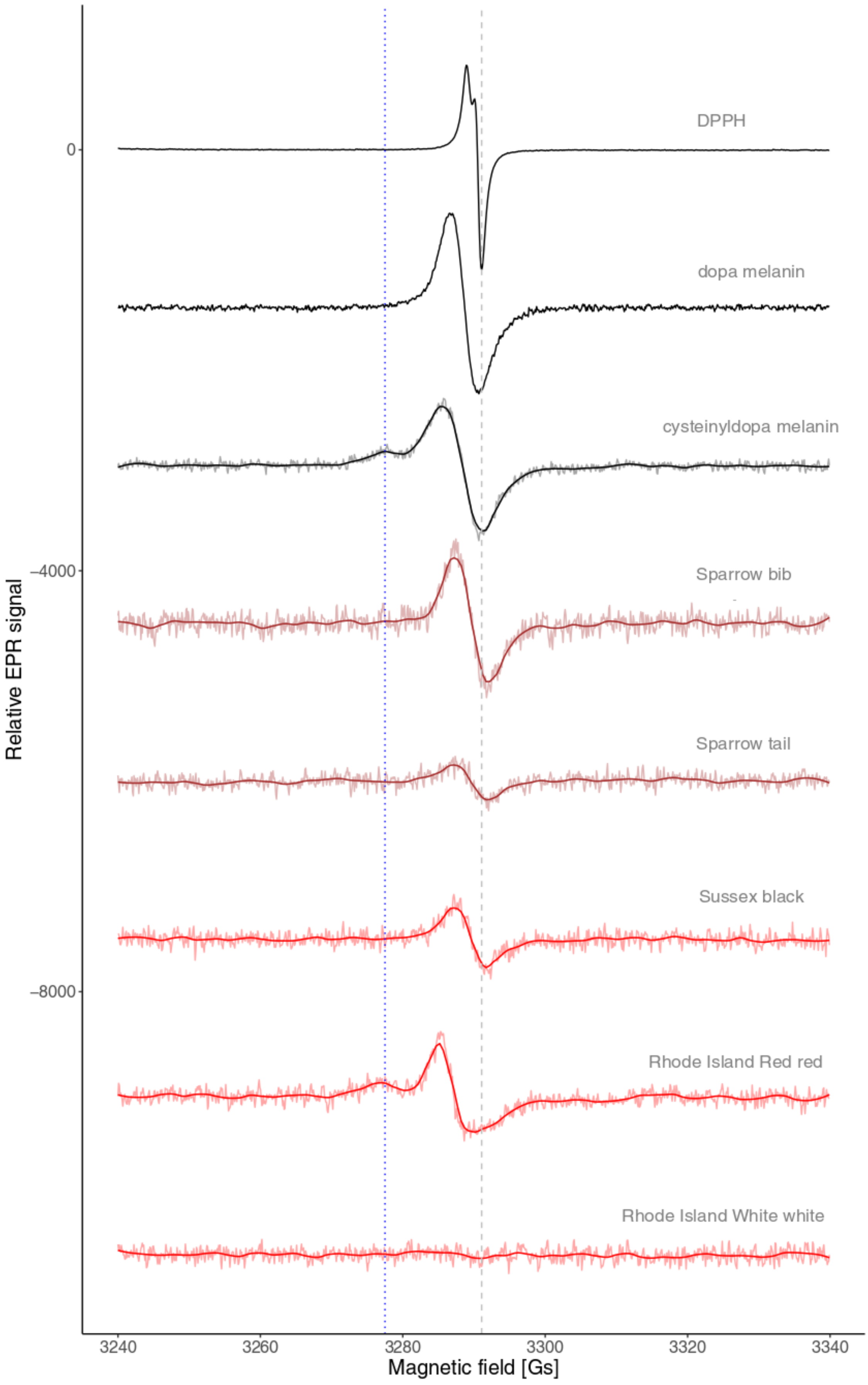

DPPH
dopa melanin
cysteinyldopa melanin
Sparrow bib
Sparrow tail
Sussex black
Rhode Island Red red
Rhode Island White white
0
−4000
−8000
Relative EPR signal
3240
3260
3280
3300
3320
3340
Magnetic field [Gs]

S2. Samples of EPR spectra of 1,1-diphenyl-2-picrylhydrazyl (DPPH; used as a paramagnetic reference), synthetic standards of eumelanins (dopa melanin), synthetic standards of pheomelanins (cysteinyldopa melanin), black bib feathers of house sparrow, brownish tail feathers of house sparrow, chicken black feather as an example of feathers containing eumelanin (Sussex black), chicken mahogany feather as an example of feathers containing pheomelanin (Rhode Island Red red), chicken white feather as an example of amelanotic feather (Rhode Island White white).

S3. Results of the model showing effects of body mass changes over the course of the experiment. The model included time, treatment (diet and injection) as fixed factors and the two-way interaction time∗diet. Reference levels for fixed effects: time: before experiment; diet: control; injection: LPS.

	Estimate	SE	*t*	*p*
Intercept	26.48	0.35	74.91	<0.001
Time (after experiment)	0.97	0.19	5.17	<0.001
Diet (PT-reduced)	-0.01	0.41	-0.02	0.99
Injection (saline)	0.38	0.39	0.99	0.33
Time (after experiment) * diet (PT-reduced)	0.62	0.26	2.36	0.02

S4. Results of the model showing effects of dietary manipulation and immune challenge on the brightness of the bib feathers. The model included time, treatment (diet and injection) as fixed factors and covariate (centered) density of feathers. Reference levels for fixed effects: diet: control; injection: LPS.

	Estimate	SE	*t*	*p*
Intercept	1208.23	80.04	15.10	<0.001
Diet (PT-reduced)	183.36	91.40	2.01	<0.05
Injection (saline)	-47.75	91.06	-0.52	0.60
density of feathers	165.05	226.35	0.73	0.47

S5. Results of the model showing effects of dietary manipulation and immune challenge on the melanin content of the bib feathers. The model included time, treatment (diet and injection) as fixed factors and covariate (centered) density of feathers. Reference levels for fixed effects: diet: control; injection: LPS.

	Estimate	SE	*t*	*p*
Intercept	16.91	0.50	33.60	<0.001
Diet (PT-reduced)	-3.15	0.59	-5.33	<0.001
Injection (saline)	0.24	0.60	0.40	0.69
density of feathers	-0.10	1.45	-0.69	0.49

S6. Results of the model showing effects of dietary manipulation and immune challenge on the melanosome density of the bib feathers. The model included treatment (diet and injection) as fixed factors and scrap ID as a random effect. Reference levels for fixed effects: diet: control; injection: LPS.

	Estimate	SE	*t*	*p*
Intercept	0.72	0.06	12.73	<0.001
Diet (PT-reduced)	0.03	0.06	0.53	0.60
Injection (saline)	-0.10	0.06	-1.70	0.10
Scrap ID	-0.001	0.004	-0.25	0.80

S7. Results of the model showing effects of dietary manipulation and immune challenge on the feathers development. The model included treatment (diet and injection) as fixed factors. Reference levels for fixed effects: diet: control; injection: LPS.

	Estimate	SE	*t*	*p*
Intercept	0.60	0.02	38.11	<0.001
Diet (PT-reduced)	-0.05	0.02	-2.84	0.006
Injection (saline)	0.04	0.02	2.47	0.02

S8. Results of the model showing effects of dietary manipulation and immune challenge on the density of the bib feathers (number of barbs). The model included treatment (diet and injection) as fixed factors. Reference levels for fixed effects: diet: control; injection: LPS.

	Estimate	SE	*t*	*p*
Intercept	1.59	0.05	34.76	<0.001
Diet (PT-reduced)	0.05	0.05	0.10	0.32
Injection (saline)	0.04	0.05	0.78	0.44
